# Baseline carcinoembryonic antigen (CEA) serum levels predict bevacizumab-based treatment response in metastatic colorectal cancer

**DOI:** 10.1111/cas.12451

**Published:** 2014-07-31

**Authors:** Gerald W Prager, Kira H Braemswig, Alexandra Martel, Matthias Unseld, Georg Heinze, Thomas Brodowicz, Werner Scheithauer, Gabriela Kornek, Christoph C Zielinski

**Affiliations:** 1 Clinical Division of Oncology, Department of Medicine I and Comprehensive Cancer Center, Medical University of ViennaAustria; 2 Section for Clinical Biometrics, Center for Medical Statistics, Informatics and Intelligent Systems, Medical University of ViennaAustria

**Keywords:** Angiogenesis inhibitors, bevacizumab, biological markers, carcinoembryonic antigen, colorectal cancer

## Abstract

Carcinoembryonic antigen (CEA) affects tumorigenesis by enhancing tumor cell survival and by inducing tumor angiogenesis. This study aimed to evaluate baseline CEA serum levels to predict bevacizumab-based therapy effect and survival in patients with metastatic colorectal cancer (mCRC). Two hundred and ninety eight mCRC patients receiving chemotherapy plus either bevacizumab or cetuximab were analyzed in a retrospective study. Disease control (DC), progression-free survival (PFS), and overall survival were assessed and related to pretreatment CEA serum levels. Patients with baseline CEA serum levels below the statistical median of 26.8 ng/mL (group I) were compared with patients with higher CEA levels (group II). The cetuximab-based treatment cohort was analyzed for specificity assessment of CEA to predict the anti-vascular endothelial growth factor effect in mCRC. Baseline CEA serum levels inversely correlated with therapeutic response in patients receiving bevacizumab-based treatment (disease control rate, 84% *vs* 60%), inversely correlated with median PFS leading to a median PFS benefit of 2.1 months for patients in group I when compared with group II, as well as inversely correlated with median overall survival (37.5 months *vs* 21.4 months). In an independent cohort of 129 patients treated with cetuximab-based therapy, no association of therapeutic response or PFS with CEA serum levels was found. As expected, baseline CEA levels were prognostic for mCRC. These data give first evidence that baseline serum CEA levels might constitute an important predictor for the efficacy of first-line bevacizumab-based therapy in patients with mCRC.

Previously, we found that CEA induces angiogenesis independent of VEGF. The data presented here now give first evidence that baseline serum CEA levels in patients might constitute an important predictor for the efficacy of first-line bevacizumab-based therapy for metastatic colorectal cancer.

The major angiogenic growth factor vascular endothelial growth factor (VEGF) and its receptor-2 (VEGFR-2) have become the focus of therapeutic intervention to block tumor angiogenesis, which has led to improved prognosis in many cancers including those originating from colon, lung, breast, and kidney.(1)– (3) Although this has been found beneficial for patients with particular tumors – albeit only for a limited duration of time – a substantial fraction of patients with cancer proves to be finally resistant to VEGF-based therapies.(4)– (6) This might lie in the fact that tumor angiogenesis is not only induced by VEGF, but also by a variety of other pro-angiogenic factors.(7) In this context, we recently described a hitherto unknown paracrine effect of the carcinoembryonic antigen (CEA), which induces pro-angiogenic endothelial cell behavior *in vitro* leading to enhanced tumor angiogenesis *in vivo*.(8) Notably, the CEA-induced angiogenesis was VEGF independent. Furthermore, CEA has been shown to affect tumor cell behavior by preventing apoptosis upon cell detachment, so-called anoikis, and by interfering with cell differentiation.(9,10)

Carcinoembryonic antigen, first identified in 1965 by Phil Gold and Samuel O. Freedman,(11) is aberrantly upregulated by as many as 70% of all human cancers. The measurement of serum levels of the extracellular biomarker CEA is routinely carried out for the monitoring of adenocarcinoma growth as well as efficacy of its treatment.(12) Under physiologic conditions, CEA – the product of the *CEACAM5* gene(13) – is mainly expressed on the apical surface of the gastrointestinal epithelium, and only low amounts of soluble CEA (approximately ≤5 ng/mL) can be detected in serum. It is highly upregulated by many different cancers and in >75% of patients with metastatic colorectal cancer (mCRC).(12)

As CEA affects tumor cell biology and its microenvironment, we hypothesized that CEA serum levels might predict the response to anti-VEGF treatment in mCRC exerted by the VEGF-targeting humanized mAb bevacizumab. Although the antibody has been approved by the FDA in 2004 for the treatment of mCRC when combined with chemotherapy, so far no validated predictive factors for VEGF-targeted therapies have been identified.(14) For this purpose, we retrospectively correlated baseline serum CEA levels with disease stabilization rates (DC), progression-free survival (PFS), as well as overall survival (OS) in mCRC patients treated with bevacizumab-based first-line therapy or, for the control, in mCRC patients treated with cetuximab-based first-line therapy.

## Materials and Methods

### Study design and patients

One hundred and sixty nine patients with mCRC treated at our center with a bevacizumab-based therapy were included in this study, all of whom met the eligibility criteria: ≥18 years old; histologically confirmed adenocarcinoma of the colon or rectum; metastatic disease unsuitable for resection with curative intent; an Eastern Cooperative Oncology Group performance status <2; and adequate organ function. The patients from our center received anti-angiogenic therapy with bevacizumab (7.5 mg/kg every 21 days or 5.0 mg/kg every 14 days) plus standard chemotherapy. The chemotherapy consisted of fluorouracil and leucovorin or capecitabine in combination with either oxaliplatin (FOLFOX, XELOX) or irinotecan (FOLFIRI, XELIRI), or capecitabine alone (1250 mg/m² b.i.d., days 1–14, every 3 weeks) at the oncologists' discretion, treated from October 2004 to December 2009. Patients had to be naive to anti-angiogenic therapies. The control cohort consisted of 129 patients with mCRC treated with cetuximab (400 mg/m^2^ baseline infusion on day 1 followed by 250 mg/m^2^ weekly) plus chemotherapy (FOLFOX6 or FOLFIRI) as previously published.(15) The presence of *KRAS* mutations in codons 12 and 13 was determined by allele-specific real-time PCR assays using validated methodology (DxS Ltd, Manchester, UK).(15) This cohort was analyzed to assess the specificity of the predictive value of CEA for bevacizumab-based treatment regiments in mCRC.

### Carcinoembryonic antigen level assessment

Carcinoembryonic antigen baseline serum levels of patients with metastatic colorectal cancer were centrally determined within 2 weeks before the first cycle of bevacizumab-based treatment. The CEA serum levels were measured with an Elecsys CEA electrochemiluminescence assay on an Elecsys 2010 system (Roche Diagnostics, Mannheim, Germany) and results were given as ng/mL.

### Assessment of response

Assessment of response was determined according to the revised Response Evaluation Criteria in Solid Tumours (RECIST) 1.1 criteria.(16) Disease control rate was defined as the proportion of subjects with best overall response, defined as either complete response, partial response, or stable disease after 10 weeks minimum time from baseline.

### Statistical methods

Objective treatment response was estimated and associated exact two-sided 95% confidence limits (Clopper–Pearson) were calculated. The time to progression or death was defined as the time from randomization until the first observation of disease progression or death due to any cause (whichever occurred earlier). If a patient had no progression at the last follow-up visit or the death date was beyond the last tumor assessment, time to progression/death was censored on the date of last tumor assessment or randomization (in case of no post-baseline tumor assessment). Overall survival was calculated from randomization until death. For patients alive at the time of analysis or lost to follow-up, data were censored at the time the patient was last determined to be alive.

Many experts discourage from categorizing continuous variables such as the baseline serum CEA level before further statistical analysis.(17) Therefore, we have depicted the hazard of progression at various CEA levels by Cox regression based on fractional polynomials of CEA, which allows for a non-linear dependency of the progression hazard on the baseline CEA level.(18,19) Point-wise 95% confidence intervals (CI) for the relative hazard have been calculated by bootstrap resampling with 1000 repetitions. However, for description by cumulative survival rates and objective treatment response rates, we have stratified our study population at the sample median of the bevacizumab group, which was 26.85 ng/mL.

Response rates were compared between groups based on the baseline serum CEA level using the χ^2^-test.

Progression-free survival, given as the percentages of subjects being alive and free of progression using RECIST criteria, were calculated using the Kaplan–Meier method.(20) The 95% CI are based on standard errors obtained by Greenwood's formula. Groups were compared by the log–rank test. Similar methods were applied to describe and compare the overall survival of the patients. All significance tests were performed two-sided at a significance level of 0.05. The statistical software system R (R development core team, Vienna, 2009; http://www.r-project.org) was used for statistical analysis.

## Results

### Patients' characteristics

One hundred and sixty nine patients with mCRC received first-line bevacizumab-based treatment. The characteristics of these patients are described in Table 1. Carcinoembryonic antigen did not show any correlation with T or N scoring (Spearman's rho = 0.01, *P* = 0.934 and rho = 0.03, *P* = 0.752, respectively). For descriptive purposes, we divided the patients into those with CEA levels at or below the median of 26.8 ng/mL (group I) and those above the median (group II). To assess the specificity of the findings for bevacizumab-based treatment, 129 mCRC patients treated with cetuximab-based regimens were analyzed.

**Table 1:** Characteristics of patients with metastatic colorectal cancer treated with bevacizumab, at baseline

Variable	Bevacizumab
*n*	169
Sex	
Female, *n* (%)	74 (43.8)
Male, *n* (%)	95 (56.2)
Age, years	
Median (quartiles)	63 (58, 67)
CEA, ng/mL	
Median (quartiles)	26.8 (5.4, 120.3)
Primary tumor location, *n* (%)	
Colon	89 (50.6)
Rectum	42 (23.9)
N/A	38 (25.5)
Metastatic sites, *n* (%)	
Intestine/bowel	7 (4.1)
Liver	127 (75.1)
Lung	65 (38.5)
Lymph nodes	22 (13.0)
Bone	5 (2.9)
T stage, *n* (%)	
1	4 (5.4)
2	10 (10.6)
3	58 (61.7)
4	21 (22.3)
Unknown	75
N stage, *n* (%)	
0	21 (23.3)
1	34 (37.8)
2	34 (37.8)
3	1 (1.1)
Missing	79

In patients with >1 metastasis per organ site, the organ site was counted once only. CEA, Carcinoembryonic antigen; N/A, not assessed

### Carcinoembryonic antigen and DC

**Fig. 1:**
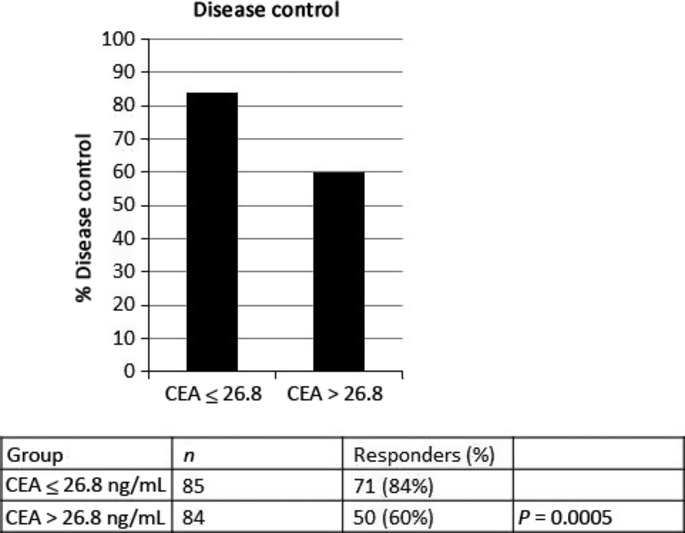
Carcinoembryonic antigen (CEA) serum levels and treatment response in patients with metastatic colorectal cancer receiving bevacizumab-based therapies. Disease control was defined as complete remission, partial remission, or stable disease.

The DC assessed according to RECIST criteria in bevacizumab-treated patients correlated with CEA serum levels: DC occurred in 71 (84%) of the 85 patients in group I, and in 50 (60%) of the 84 patients in group II (χ^2^-test, i < 0.005: odds ratio, 0.579; 95% CI, 0.422–0.795; *P* < 0.001) (Fig. 1).

### Carcinoembryonic antigen and PFS

**Fig. 2:**
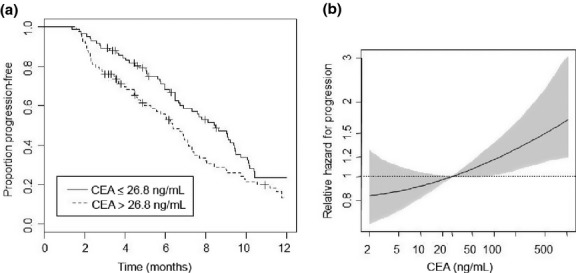
Bevacizumab-based therapies and progression-free survival (PFS) in patients with metastatic colorectal cancer. (a) Kaplan–Meier estimates for PFS. Subgroups according to the baseline serum carcinoembryonic antigen (CEA) levels, categorized at the observed median of 26.8 ng/mL. (b) Hazard of progression *versus* baseline CEA serum level, relative to the median baseline serum CEA level (26.8 ng/mL) with point-wise 95% confidence intervals.

As shown in Figure 2 and Table 2, the median PFS for patients receiving bevacizumab-based treatment was 8.5 months in group I and 6.4 months in group II (*P* = 0.023). One-year PFS rates were 24% (95% CI 15–36%) in group I, and 13% (95% CI 7–25%) in group II (Table 2, Fig. 2a). The hazard ratio (HR) of group II *versus* group I was 1.47 (95% CI, 1.05–2.05). This HR was virtually unchanged if adjusted for T and N (HR, 1.47; 95% CI, 1.04–2.09). Figure 2(b) depicts the hazard of progression at various baseline serum CEA levels, relative to the median level of 26.8 ng/mL. This analysis indicates the almost linear dependency of the progression risk on the baseline serum CEA level. Compared to the median level of 26.8 ng/mL, patients with CEA levels of >69 ng/mL were at a significantly elevated risk for progression. In contrast, the progression risk appeared to be lower for patients with CEA levels lower than 26.8 ng/mL, but a significance level was not reached.

**Table 2:** Progression-free survival (PFS) and overall survival (OS) patients with metastatic colorectal cancer treated with bevacizumab, grouped according to serum carcinoembryonic antigen (CEA) level

PFS in CEA populations		
Median survival, months	Bevacizumab	
CEA ≤26.8 ng/mL	8.5 months	
CEA >26.8 ng/mL	6.4 months	
*P*-value in group	0.023	
Survival rate in per cent (95% CI)	CEA≤26.8 ng/mL	CEA >26.8 ng/mL
3 months	89 (83–96)	76 (68–86)
6 months	68 (59–79)	54 (44–67)
9 months	47 (37–60)	27 (19–40)
12 months	24 (15–36)	13 (7–25)
T stage, hazard ratio (95% CI)	0.89 (0.66–1.22)	
	*P* = 0.465	
N stage, hazard ratio (95% CI)	1.18 (0.85–1.62)	
	*P* = 0.325	

OS in CEA populations		
Median survival, months	Bevacizumab	
CEA ≤26.8 ng/mL	37.5 months	
CEA >26.8 ng/mL	21.4 months	
*P*-value in group	0.019	
Survival rate in per cent (95% CI)	CEA ≤26.8 ng/mL	CEA >26.8 ng/mL
6 months	92 (86–98)	89 (82–96)
12 months	81 (73–90)	68 (58–79)
18 months	69 (59–81)	57 (47–70)
24 months	62 (51–75)	43 (32–58)
T stage, hazard ratio (95% CI)	1.05 (0.70–1.58)	
	*P* = 0.803	
N stage, hazard ratio (95% CI)	1.46 (0.97–2.20)	
	*P* = 0.068	

Median time and survival rates are based on Kaplan–Meier estimates. CI, confidence interval.

### Carcinoembryonic antigen and OS

**Fig. 3:**
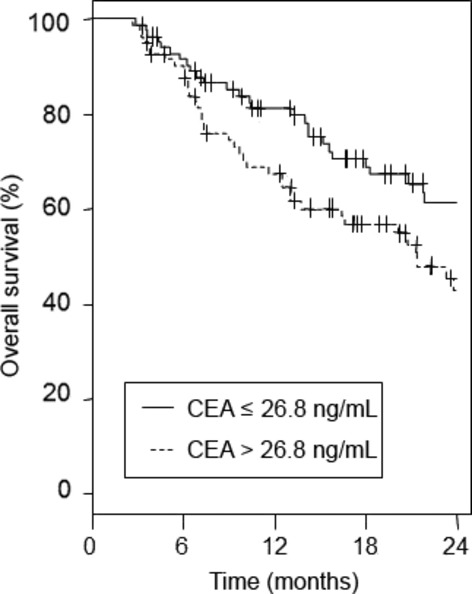
Kaplan–Meier estimates for overall survival in patients with metastatic colorectal cancer treated with bevacizumab, grouped according to baseline serum carcinoembryonic antigen (CEA) level (CEA ≤26.8 ng/mL *vs* CEA >26.8 ng/mL).

Median OS was 37.5 months in group I and 21.4 months in group II (*P* = 0.019). The survival rates at 24 months were 62% (95% CI, 51–75%) and 43% (95% CI, 32–58%), respectively (Fig. 3a). The HR of group II *versus* group I was 1.71 (1.08–2.70). This HR was virtually unchanged if adjusted for T and N (HR, 1.78; 95% CI, 1.11–2.85).

One hundred and eighteen patients (69.8%) were assessable for analysis of subsequent treatments, whereby available data were balanced between the low and high baseline CEA groups (58/118 [49.15%] and 60/118 [50.85%], respectively). In both groups, anti-EGFR antibodies (cetuximab or panitumumab) were given in further line treatments (low CEA group, 41.37%; high CEA group, 36.67%). No significant differences were found in the number of subsequent treatment lines between low and high baseline CEA patients: 31.0% and 10.3% of the low CEA patients and 26.7% and 10.0% of the high CEA patient group received a third- and ≥fourth-line therapy. Thus, subsequent treatment did not significantly affect the prognostic value of CEA (*P* = 0.56).

### Baseline CEA levels and cetuximab-based therapies in mCRC

**Fig. 4:**
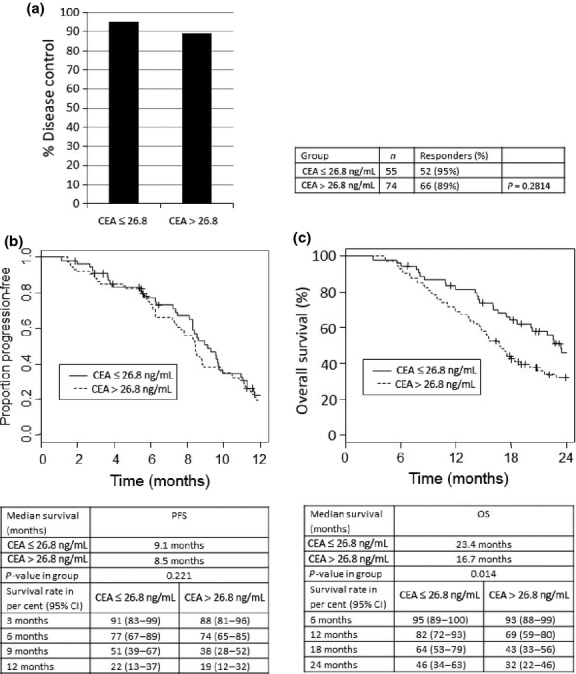
Baseline serum carcinoembryonic antigen (CEA) levels and cetuximab-based therapies in patients with metastatic colorectal cancer. (a) Disease control was defined as complete remission, partial remission, or stable disease; CEA groups in the cetuximab population according baseline serum CEA (≤26.8 ng/mL *vs* >26.8 ng/mL). (b) Kaplan–Meier estimates for progression-free survival (PFS). Subgroups according to the baseline serum CEA levels, categorized at 26.8 ng/mL. (c) Kaplan–Meier estimates for overall survival (OS) by CEA group in cetuximab population (CEA ≤26.8 ng/mL *vs* CEA >26.8 ng/mL).

Several reports have described that elevated CEA baseline levels might be prognostic.(21,22) Consistent with these results, the current study confirms a prognostic role of baseline CEA levels in mCRC patients treated with anti-VEGF-based therapy. In order to analyze whether baseline CEA serum levels simply reflect high tumor load, resulting in detrimental treatment outcome, a separate cohort of 129 patients who received cetuximab-based chemotherapy was analyzed. Thereby, baseline CEA serum levels (categorized at 26.8 ng/mL) were not associated with either DC (*P* = 0.281; Fig. 4a) or PFS (*P* = 0.221; Fig. 4b). However, as expected, CEA was prognostic as the OS in cetuximab-treated patients with higher CEA levels was decreased (median survival 23.4 months for group I *vs* 16.7 months for group II, *P* = 0.014; Fig. 4c).

## Discussion

Etiological concepts on cancer development, malignant growth, and tumor propagation have undergone a revolutionary development during recent years. Among other aspects, the discovery of angiogenesis, the growth of new blood vessels from preexisting vasculature, as a key element in the pathogenesis of malignancy(7) has opened an abundance of biologic insights and subsequent therapeutic options, which have led to improved prognosis in many cancers including those originating from colon, lung, breast, and kidney. As VEGF represents the main pro-angiogenic stimulator,(23) it is currently in focus for therapeutic interventions and has resulted in the registration of bevacizumab by the FDA and *European Medicines Agency* (EMA) for the treatment of mCRC(24) and other malignancies. Nevertheless, the use of VEGF-targeting drugs has been shown to be only effective for certain patients, who eventually become resistant to these drugs.(5,6) However, indications exist that certain subgroups of patients might benefit from long-term bevacizumab therapies.(25) Thus, continuing attempts have aimed to characterize biomarkers indicative for bevacizumab efficacy in certain patient populations ultimately resulting in targeted treatment strategies. This would not only ameliorate overall treatment results, but also help in cost reduction when treating patient populations with a certain disease entity. Only a few biomarkers such as angiopoietin-2,(26) circulating levels of short VEGF-A isoforms, soluble VEGFR-1,(27) or intramural expression of VEGFR-2 or neuropilins(28) have so far been proposed. However, theses markers need to be validated.

As new therapeutic options emerge, it is desirable to obtain increased insights in molecular and tumor cell biology to optimize and individualize therapy.(29) In this context, CEA has been shown to affect tumor cell biology in an autocrine manner, leading to increased cell survival and inhibition of tumor cell differentiation.(30) Consistently, it was recently shown that CEA injection led to an increase in metastasis formation in mice.(31) In addition, we have previously observed a novel functional role of CEA in endothelial cell activation and tumor angiogenesis, effects that were induced in a paracrine manner.(8) Thereby, CEA acted independently of the major angiogenic growth factor VEGF. It was, therefore, tempting to investigate whether CEA levels predict treatment effects in anti-VEGF-based therapies. To address this assumption, we have retrospectively analyzed the impact of initial CEA serum levels on the clinical outcome of patients with mCRC treated in our center with bevacizumab-based therapy combined with cytotoxic drugs by a treatment interaction design.

So far, baseline CEA has only been identified to be a prognostic marker in metastasized and non-metastasized CRC.(21,32) This effect might partially be explained by the fact that high tumor load is accompanied with higher CEA serum levels. That baseline CEA levels are associated with prognosis was consistently observed in our study; thereby, baseline CEA levels were prognostic independent of the targeted treatment, in anti-VEGF- as well as in anti- EGFR-treated patients. To exclude that high tumor volume, however, is responsible for the treatment efficacy in mCRC, we included a control cohort of mCRC patients with comparable baseline characteristics. The cetuximab-based cohort, published in a previous communication,(15) was evaluated for CEA and treatment efficacy and compared to the bevacizumab cohort. The negative results in the control cohort revealed that baseline CEA levels were only predictive for bevacizumab-based treatment, suggesting CEA as a specific marker of response and PFS for anti-VEGF treatment in mCRC. A treatment cohort characterized by chemotherapy alone with lack of an antibody addition was not available in our center, because targeted therapies in the first-line setting have been widely used since 2005. Patients treated before 2005 received different chemotherapy treatment regimens including bolus 5-fluouracil, which prevented these patients forming a control group. The control group used in our study, cetuximab plus chemotherapy, also had some limitations as in this group every patient received a doublet chemotherapy as a backbone to anti-EGFR. In the bevacizumab cohort, 39% had fluoropyrimidine alone plus bevacizumab, which might explain the difference in PFS. However, this did not affect the predictive value of baseline CEA in the bevacizumab cohort.

In our study, we used a categorization of CEA levels at the statistical median of 26.8 ng/mL for descriptive purposes. In the present analysis, a statistical median level of CEA 26.8 ng/mL was the cut-off for bevacizumab-responders with a favorable prognosis for patients with a CEA level below 26.8 ng/mL. However, analysis of the progression risk with CEA as a continuous variable did not suggest a natural candidate cut-off level. Rather, the progression risk was continuously increasing with increasing CEA levels. Although our study has some methodological limitations, the data suggest that CEA as a classic biomarker with intrinsic activity on tumor cells and tumor microenvironment might predict therapeutic response towards anti-VEGF therapy in mCRC.

As we have recently shown that CEA is capable of inducing endothelial cell activation in the presence of VEGF inhibitors,(8) it is tempting to speculate that high CEA levels might functionally bypass anti-VEGF antibody treatment regimens. Although not shown here, our data on baseline CEA levels in predicting treatment response towards anti-VEGF are consistent with this hypothesis. Expression of CEA can be induced by inflammation or tobacco smoking, however, serum levels observed under these conditions do not usually exceed 5 ng/mL, which makes it unlikely that this affects the predictive value of CEA in anti-VEGF treatment.

The data presented here provide the first evidence that the canonical biomarker CEA, which is used for monitoring tumor growth of adenocarcinomas as well as treatment efficacy, might have not only a biological role, but could provide a predictive function for anti-VEGF-based combination therapies. The described effects of CEA on treatment outcome were obviously limited to bevacizumab-based treatment and were independent from various degrees of metastatic spread, as observed in a separately analyzed cohort of 129 patients who received cetuximab-based chemotherapy as a first-line treatment.

We thus conclude that baseline CEA serum levels might represent a first step in optimizing and individualizing anti-angiogenic therapies with bevacizumab-based treatment in mCRC to maximize patient benefit and prevent an ineffective treatment with potential side-effects. Following the present retrospective analysis, further studies to replicate our findings as well as a prospective validation are needed to finally translate this marker into clinical practice.
